# Driving Impairment and Healthcare Provider Counseling in Rheumatoid Arthritis and Osteoarthritis Patients: A Cross-Sectional Survey

**DOI:** 10.7759/cureus.26280

**Published:** 2022-06-24

**Authors:** Cristina E Romaniello, Anna C Falls, PetaGay Ricketts, Amulya Dega, John O Elliott, Kim M Jordan

**Affiliations:** 1 Department of Internal Medicine, OhioHealth Riverside Methodist Hospital, Columbus, USA; 2 Division of Immunology, Allergy, and Rheumatology, University of Cincinnati Medical Center, Cincinnati, USA; 3 Division of Rheumatology and Clinical Immunology, University of Pittsburgh Medical Center, Pittsburgh, USA; 4 Department of Internal Medicine and Graduate Medical Education, OhioHealth, Columbus, USA; 5 Department of Medical Education, OhioHealth Riverside Methodist Hospital, Columbus, USA

**Keywords:** rheumatoid arthritis, haq-di, iadl (instrumental activities of daily living), safety, physician counseling, driving, osteoarthritis

## Abstract

Background/objective: To examine rates of counseling on driving for individuals with osteoarthritis (OA) and/or rheumatoid arthritis (RA) and evaluate the Health Assessment Questionnaire Disability Index (HAQ-DI) as a screening tool for further driving evaluation.

Methods: A cross-sectional survey was completed by individuals recruited via ResearchMatch (a national web-based recruitment tool) between March 5 and April 20, 2020. Individuals with a current US driver's license, ≥18 years old, with self-reported OA and/or RA diagnosis were surveyed about driving difficulty and vehicle modification and completed a HAQ-DI assessment. Respondents were dichotomized based on reporting vehicle modification(s) due to arthritis versus no modification(s) for univariate and multivariate analyses.

Results: Of 4,435 recruited patients, 304 (6.9%) met inclusion/exclusion criteria and completed the surveys. Of all respondents, 259 (85.2%) reported at least some difficulty with one or more driving activities, but only 47 (15.5%) reported discussion with a physician and/or healthcare professional. A total of 184 (60.5%) respondents had HAQ-DI ≥ 1 and were more likely to report vehicle modification(s) compared to respondents with HAQ-DI score < 1 (OR = 5.00, 95% CI = 2.69-9.32, p < 0.011) after controlling for age, gender, type of arthritis, and driving behaviors.

Conclusion: Few respondents report discussion of driving difficulties with healthcare providers, although many report driving-related impairments, particularly those with HAQ-DI scores ≥ 1. Our data suggest a strong association between HAQ-DI scores and vehicle modification. The HAQ-DI may serve as a screening tool to predict a patient’s need for driving evaluation and vehicle modification(s).

## Introduction

Osteoarthritis (OA) and rheumatoid arthritis (RA) cause functional impairments that impact daily life activities, including automobile driving. Radiographic presence of OA on the knee and hip imaging significantly affects braking response time, and RA often causes impairment in hand and upper extremity functions in driving [1-5]. Patients with RA often require vehicle modifications to operate a vehicle safely and comfortably [1,4-9]. Analysis of data from the Second Strategic Highway Research Program (SHRP 2) reported that “drivers with arthritis were 72% (OR 1.72) more likely to be involved in a motor vehicle crash,” when accounting for age [10].

Few studies have evaluated rates of physician counseling on driving difficulties or risks in this patient population, but reported rates are low, ranging from 0% to 8% [5,9,11]. Dawson et al. reported only 24% of surveyed patients with RA directly sought driving counseling, and Vrkljan et al. reported that patients may be hesitant to seek advice due to concern of driver’s license revocation [11,12].

In the United States, rates of physician counseling on driving safety in OA and/or RA patients are unknown. Furthermore, limited studies address the association between functional disability scores, particularly the Health Assessment Questionnaire Disability Index (HAQ-DI), with driving impairment or the need for vehicle modifications in patients with OA and/or RA [5,6,9]. Reports have correlated a HAQ-DI score ≥ 1 to driving difficulty, but driving difficulty was also present in patients with lower scores [5,9]. Zhou et al. emphasized the need for further research in driving safety and vehicle adaptations in the first systematic review addressing driving ability and safety in RA [8].

To address existing information gaps, we surveyed patients with self-reported diagnoses of OA and/or RA on functional disability (HAQ-DI), driving habits and limitations, use of vehicle modifications, and whether they received counseling about driving safety by a healthcare professional. Our secondary goal was to evaluate HAQ-DI as a possible screening tool to assist and identify patients needing driving counseling.

## Materials and methods

The study was designed as a cross-sectional survey and received Institutional Review Board approval. Participants were recruited through the ResearchMatch network, a national web-based recruitment tool maintained at Vanderbilt University Medical Center. ResearchMatch consists of a registry of volunteers who sign up to receive notifications about potential research studies. A study-specific recruitment message was electronically sent to 4,435 potential matches. If individuals chose to participate, they were re-directed to electronic inclusion/exclusion questions, a study-specific survey (Appendix), and the HAQ-DI. The survey was available to participants from March 5 to April 20, 2020, with two reminders for completion sent during this period.

Volunteers met inclusion/exclusion criteria if they held a current United States driver’s license, were at least 18 years old, and had a self-reported diagnosis of RA, OA, or cervical spinal fusion or joint replacement (hip, knee, or shoulder) due to arthritis. Based on current literature, a study-specific survey was developed that collected demographic information, current driving habits and limitations, use of vehicle modifications, and whether driving counseling had occurred. All responses were securely maintained on our institution’s electronic REDCap database (Vanderbilt University, Nashville, TN).

The HAQ-DI is a self-reported assessment of participants’ functional status (dressing, arising, eating, walking, hygiene, reach, grip, and common daily activities). Total scores and various subscales were calculated using the scoring instructions, which yield a standardized score ranging from 0 (no disability) to 3 (severe disability). The reliability and validity of the HAQ-DI have been previously established [13]. Based on previously reported research [9], we dichotomized the HAQ-DI using a score ≥ 1 as an indicator of moderate to severe disability and scores < 1 as an indicator of less than moderate disability.

The dependent variable for the study was defined based on respondents reporting one or more vehicle modification(s) versus no modification. This was based on the following survey question: “What modifications have you made to your vehicle because of your arthritis? Check all that apply. You may have more than one answer: seat supports, extra mirrors, padded steering wheel, hand brake, remote start.”

Statistical analyses

First, univariate analyses (chi-square and independent samples t-tests) were conducted. If Levene’s test for equality of variances was significant, then non-parametric Wilcoxon rank-sum tests were conducted. Next, logistic regression analyses were conducted to control for participant characteristics including age, gender, arthritis diagnosis questions, miles driven, roads, and car type. All items were entered into a logistic model to determine if a smaller number of individual HAQ-DI questions could be utilized as a screen. Statistical significance was based on traditional two-sided tests with an alpha error of 5%. Statistical analyses were conducted using IBM SPSS Statistics version 25 (IBM Corp., Armonk, NY).

## Results

Of the 4,435 volunteers, 356 (8.0%) agreed to be contacted, 324 (7.3%) participated, and 304 (6.9%) met the inclusion/exclusion criteria and completed the survey. A total of 198 respondents reported OA, 51 reported RA, and 55 reported both OA and RA. A total of 105 respondents reported one or more vehicle modifications were made due to arthritis, and 199 reported no modification(s). There were no differences in demographic characteristics, self-reported diagnoses, and current driving patterns (Table 1).

**Table 1:** Participant characteristics Notes: The questions on knee replacement, hip replacement, shoulder replacement, and spinal fusion were all asked with “due to arthritis.” Outcome defined based on the question: “What modifications have you made to your vehicle because of your arthritis? Check all that apply. You may have more than one answer: seat supports, extra mirrors, padded steering wheel, hand brake, remote start.”

	Respondent demographics		
Characteristic	Any (n = 105)	None (n = 199)	P-value
Age, mean age ± standard deviation	56.4 ± 12.5	59.4 ± 12.0	0.052
Gender, % (n)			0.204
Male	12.4% (13)	19.7% (39)	
Female	87.6% (92)	79.2% (158)	
Other	0% (0)	0.5% (1)	
Diagnosis, % (n)			
Osteoarthritis	84.8% (89)	82.4% (164)	0.633
Rheumatoid arthritis	30.5% (32)	37.2% (74)	0.257
Knee replacement	15.2% (16)	18.6% (37)	0.527
Hip replacement	9.5% (10)	11.6% (23)	0.700
Shoulder replacement	0% (0)	3.5% (7)	0.100
Spinal fusion	9.5% (10)	10.6% (21)	0.844
	Driving characteristics		
Current driver, % (n)	98.1% (103)	99.0% (197)	0.611
Miles driven per day, % (n)			0.524
Less than 10	43.8% (46)	42.2% (84)	
10-20	34.2% (36)	30.2% (60)	
More than 20	21.9% (23)	27.6% (55)	
Type of roads driven, % (n)			0.290
Two-lane	13.3% (14)	20.6% (41)	
Four-lane	4.8% (5)	4.0% (8)	
Both	81.9% (86)	75.4% (150)	
Vehicle type, % (n)			0.590
Automatic	96.2% (101)	94.5% (188)	
Manual	3.8% (4)	5.5% (11)	

Respondents who reported vehicle modification(s) were more likely to report a prior car accident due to their arthritis and reported significant difficulty with all driving activities (Table 2). Of all participants, 6.9% reported receiving driving counseling by physicians (8.6% of those with vehicle modifications and 6.1% of those with no vehicle modifications). Of all participants, 8.6% reported receiving driving counseling by another healthcare professional (11.4% of those with vehicle modifications and 7.1% of those with no vehicle modifications). Only one participant reported a referral for a formal driving evaluation.

**Table 2:** Healthcare professional discussion and participants' driving difficulties % = percent of respondents; n: number of respondents.

	Vehicle modifications		
Characteristics	Any (n = 105)	None (n = 199)	P-value
Have you ever had a car accident or fender bender because of difficulties from your arthritis?	10.6% (11)	2.5% (5)	0.004
Has a physician ever asked you about driving difficulties related to your arthritis?	8.6% (9)	6.1% (12)	0.477
If yes, did he or she provide you with advice and resources to help with your driving difficulties?	0% (0)	0.5% (1)	1.000
Has any other medical professional (i.e., medical assistant, nurse, physician assistant or nurse practitioner, physical or occupational therapist, or chiropractor) asked you about driving difficulties related to your arthritis?	11.4% (12)	7.1% (14)	0.205
Beyond your initial testing for a driver’s license, have you ever been referred for a driver's evaluation because of your arthritis?	0% (0)	0.5% (1)	1.000
Because of your arthritis, how much difficulty do you have with each of the following activities? % (n) “With SOME/MUCH difficulty/Unable to do”			
Getting in and out of a car	87.6% (92)	66.7% (132)	<0.001
Reversing or backing up the car	62.5% (65)	40.1% (79)	<0.001
Gripping the steering wheel	43.8% (46)	24.0% (47)	0.001
Turning the steering wheel	41.3% (43)	23.2% (45)	0.001
Turning the ignition	20.0% (21)	7.1% (14)	0.001
Acceleration	20.0% (21)	7.1 % (14)	0.001
Braking	27.9% (29)	10.2% (20)	<0.001
Shifting gears	25.0% (26)	9.8% (19)	0.001

Participants who reported vehicle modification(s) indicated greater than moderate disability, based on HAQ-DI scores ≥ 1, as well as HAQ-DI subscales (Table 3). They also reported higher pain scores in the past week and poorer functioning based on the two visual analog scales of the HAQ-DI.

**Table 3:** Participants' HAQ-DI scores HAQ-DI: Health Assessment Questionnaire Disability Index; VAS: visual analog scale; n: number of respondents.

	Vehicle modifications		
Characteristic	Any (n = 105)	None (n = 199)	P-value
HAQ-DI standardized score, mean ± standard deviation	1.34 ± 0.53	0.90 ± 0.54	<0.001
HAQ-DI score ≥ 1, % (n)	81.0% (85)	49.7% (99)	<0.001
HAQ-DI subscales			
Dress	1.02 ± 0.69	0.60 ± 0.67	<0.001
Arise	1.24 ± 0.64	0.78 ± 0.65	<0.001
Eat	0.88 ± 0.72	0.50 ± 0.68	<0.001
Walk	1.31 ± 0.86	0.99 ± 0.79	0.002
Hygiene	1.65 ± 1.00	1.19 ± 1.05	<0.001
Reach	1.54 ± 0.80	1.02 ± 0.82	<0.001
Grip	1.07 ± 0.87	0.49 ± 0.74	<0.001
Activity	2.05 ± 0.63	1.60 ± 0.88	<0.001
How much pain have you had because of your illness in the past week: VAS scale 0-100 (no pain-severe pain)	59.6 ± 23.6	47.5 ± 27.9	0.001
Considering all the ways that your arthritis affects you, rate how you are doing on the following scale: VAS scale 0-100 (very well-very poor)	48.2 ± 25.1	37.7 ± 26.4	0.001

In the unadjusted logistic regression model, respondents with a score of ≥1 were significantly more likely to report any vehicle modification versus those with scores of <1 (OR = 4.29, 95% CI: 2.45-7.52, p < 0.001) with 12.9% of the variance (R^2^) in self-reported driving modification being accounted for. After controlling for age and gender (Table 4, Model 1), respondents with HAQ-DI scores ≥ 1 had increased odds of reporting any vehicle modifications (OR = 4.80, 95% CI: 2.68-8.59, p < 0.001, R^2^ = 16.2%). The odds of reporting vehicle modification further increased after controlling for all arthritis diagnosis questions (OR = 4.95, 95% CI: 2.71-9.05, p < 0.001, R^2^ = 19.2%) and when controlling for miles driven and road/car types (OR = 5.00, 95% CI: 2.69-9.32, p < 0.001, R^2^ = 20.7%).

**Table 4:** Association between HAQ-DI and self-reported driving modifications Notes: Model 1 controls for age and gender. Model 2 controls for age, gender, and arthritis diagnosis questions. Model 3 controls for age, gender, arthritis diagnosis questions, miles driven, roads, and car type. HAQ-DI: Health Assessment Questionnaire Disability Index.

	Vehicle modifications (any vs. none)		
HAQ-DI score ≥ 1 versus < 1	Odds ratio	95% CI	P-value
Unadjusted model	4.29	2.45-7.52	<0.001
Model 1	4.80	2.68-8.59	<0.001
Model 2	4.95	2.71-9.05	<0.001
Model 3	5.00	2.69-9.32	<0.001

Of all the HAQ-DI items, four had an independent association with reported vehicle modifications: two aids or devices, specifically special or built-up chairs (OR = 4.51, 95% CI: 1.41-14.41, p = 0.011) and a bathtub bar (OR = 2.59, 95% CI: 1.44-4.65, p = 0.001), one grip question, i.e., “Open jars which have been previously opened?” (OR = 2.16, 95% CI: 1.44-3.23, p < 0.001), and one activity question, i.e., “Do chores such as vacuuming or yardwork?” (OR = 1.57, 95% CI: 1.13-2.20, p = 0.008). These four items from the HAQ-DI account for 26% of the variance in self-reported driving modifications.

## Discussion

Driving is essential for patients with arthritis to continue to be active within their communities, so discussion and encouragement of safe driving practices are of great importance [12]. We found that the majority (85.2%) of our survey respondents reported some level of driving impairment, but few reported assessments of driving practices, comfort, and/or safety by a healthcare provider. Our findings of low rates of physician counseling on driving difficulties are similar to rates noted in studies in Canada and England [5,9].

A recent review emphasized the need for occupational therapists and healthcare providers to make patients aware of vehicle modifications, but many professionals report a lack of confidence or awareness in the discussion of these topics [14]. Moreover, system-level issues in the clinical setting such as time restraints, competing priorities, and lack of training may impact the frequency of conversations about driving safety and comfort [15]. In addition to time barriers, conversations regarding driving difficulties may hinder the doctor-patient relationship. Patients may battle feelings of loss of autonomy and anxiety that can present challenges to discussions about driving safety and comfort with their physician [16]. As a result, screening tools that aid healthcare providers in recognition of individuals requiring additional counseling or a more formalized assessment of driving ability or difficulty are needed. These tools could be utilized by other members of the healthcare team, such as medical assistants, registered nurses, occupational or physical therapists, or completed by patients themselves. Toolkits to aid clinicians with in-office driving counseling have previously been developed for patients with dementia, and research is ongoing to develop similar resources for patients with arthritis [12].

We propose that the HAQ-DI may be an initial screening tool to identify patients at risk for driving difficulties. Respondents with HAQ-DI ≥ 1 reported significantly greater difficulty with all surveyed activities and greater use of vehicle modifications compared to those with HAQ-DI < 1, regardless of other variables. Furthermore, driving accidents increased among those individuals who reported vehicle modifications. Thus, individuals with HAQ-D1 scores ≥ 1 may benefit from earlier counseling and more formalized driving assessment based on these increased reported rates of driving difficulties and vehicle modification(s) within this population. Our analysis of all individual HAD-QI items suggests that four items may suffice as a brief screening tool over the whole HAD-QI assessment: reported “yes” to use of a bathtub bar or a special chair, reported difficulty with opening jars (grip strength), or reported difficulty with specific chores like yard work or using a vacuum cleaner. Additional studies are required to validate the use of these four questions as a short screening tool to prompt a more formal investigation into driving difficulty and safety.

Limitations

We note several limitations to our study. Some of our questions were developed de novo, which limits validity and reliability. While ResearchMatch includes participants from around the United States, it is a voluntary organization consisting of people interested in research who have internet access, which increases response bias. Further, ResearchMatch is not a random sample of the population, which limits generalizability, and all responses are subjective self-reports with no verification of clinical diagnoses by a healthcare professional. We were unable to assess or confirm the severity of arthritic deformities. Future surveys that query medication use (for example, the use of disease-modifying anti-rheumatic drugs) may help confirm self-reported diagnoses. Lastly, our research is cross-sectional in nature so we can only present associations between variables, as cause and effect relationships cannot be tested.

## Conclusions

The low rates of counseling on driving safety and vehicle modification emphasize the need for future work to better understand and remove barriers for both physicians or other healthcare providers and patients with OA and/or RA. The HAQ-DI score (or an abbreviated form using four questions from the HAQ-DI) may be useful as an initial screening tool or used with existing toolkits to assist healthcare providers in providing needed counseling in this patient population.

## Appendices

Study-specific driving survey

1. Are you a current driver?

○ Yes ○ No

2. How many miles do you drive per day on average?

○ Less than 10 ○ 10 to 20 ○ More than 20

3. On what type of roads do you mainly drive?

○ Two-lane roads ○ Four-lane roads ○ Both two-lane and four-lane roads

4. What type of car do you mainly drive?

○ Automatic transmission ○ Manual transmission

5. How much difficulty do you have with each of the following activities?

Please choose a number from 0 to 3 for each of the activities.

0 = without ANY difficulty

1 = with SOME difficulty

2 = with MUCH difficulty

3 = unable to do

a. Getting in and out of a car: ____

b. Reversing or backing up the car: ____

c. Gripping the steering wheel: ____

d. Turning the steering wheel: ____

e. Turning the ignition: ____

f. Acceleration: ____

g. Braking: ____

h. Shifting gears: ____

6. Have you ever had a car accident or “fender bender” because of difficulties from your arthritis?

○ Yes ○ No

7. Has a physician ever asked you about driving difficulties related to your arthritis?

○ Yes ○ No

8. Has any other medical professional (i.e., medical assistant, nurse, physician assistant or nurse practitioner, physical or occupational therapist, or chiropractor) asked you about driving difficulties related to your arthritis?

○ Yes ○ No

9. Beyond your initial testing for a driver’s license, have you ever been referred for a driver’s evaluation?

○ Yes ○ No

10. Was your driving evaluation on the road or virtual (i.e., using computer simulation)?

○ On-road test

○ Virtual (computer-based) test

○ Both virtual and on road

○ Not applicable

11. What modifications have you made to your vehicle? Check all that apply. You may have more than one answer.

○ Seat supports

○ Extra mirrors

○ Padded steering wheel

○ Hand brake

○ Remote start

○ No modifications made

12. What is your age? ____ ○ Prefer not to answer

13. What is your gender?

○ Male

○ Female

○ Prefer not to answer

○ Other

## References

[1] Murray-Leslie C (1991). Driving for the person disabled by arthritis. Br J Rheumatol.

[2] von Bernstorff M, Feierabend M, Jordan M, Glatzel C, Ipach I, Hofmann UK (2017). Radiographic hip or knee osteoarthritis and the ability to drive. Orthopedics.

[3] Hofmann UK, Jordan M, Rondak I, Wolf P, Kluba T, Ipach I (2014). Osteoarthritis of the knee or hip significantly impairs driving ability (cross-sectional survey). BMC Musculoskelet Disord.

[4] Jones JG, McCann J, Lassere MN (1991). Driving and arthritis. Br J Rheumatol.

[5] Busteed S, Daly M, Silke C, Molloy MG (2004). Rheumatoid arthritis impairs driving ability even in patients with a low disability index. Rheumatology (Oxford).

[6] Katz PP, Morris A, Yelin EH (2006). Prevalence and predictors of disability in valued life activities among individuals with rheumatoid arthritis. Ann Rheum Dis.

[7] Cornwell M (1987). The assessment of people with arthritis who wish to drive a car. Int Disabil Stud.

[8] Zhou DJ, Mikuls TR, Schmidt C (2021). Driving ability and safety in rheumatoid arthritis: a systematic review. Arthritis Care Res (Hoboken).

[9] Cranney AB, Harrison A, Ruhland L (2005). Driving problems in patients with rheumatoid arthritis. J Rheumatol.

[10] Almannaa Almannaa, M. M., Bareiss Bareiss, M. M., Riexinger Riexinger, L. and Guo (2022). Are individuals with arthritis more likely to be involved in a crash?. F.

[11] Dawson JK, Campbell EA, Byrne J, Lynch MP (1995). Advice to car drivers with rheumatoid arthritis. Br J Rheumatol.

[12] Vrkljan BH, Cranney A, Worswick J (2010). Supporting safe driving with arthritis: developing a driving toolkit for clinical practice and consumer use. Am J Occup Ther.

[13] Sousa KH, Kwok OM, Ryu E, Cook SW (2008). Confirmation of the validity of the HAQ-DI in two populations living with chronic illnesses. J Nurs Meas.

[14] Cammarata M, Sangrar R, Harris JE, Richardson J, Vrkljan B (2020). A scoping review of environmental factors that impact driving with arthritis: considerations for occupational therapy. Occup Ther Health Care.

[15] Betz ME, Jones J, Petroff E, Schwartz R (2013). "I wish we could normalize driving health:" a qualitative study of clinician discussions with older drivers. J Gen Intern Med.

[16] Scott TL, Liddle J, Pachana NA, Beattie E, Mitchell GK (2020). Managing the transition to non-driving in patients with dementia in primary care settings: facilitators and barriers reported by primary care physicians. Int Psychogeriatr.

